# Association of Ambient air Pollution with risk of preeclampsia during pregnancy: a retrospective cohort study

**DOI:** 10.1186/s12889-020-09719-w

**Published:** 2020-11-05

**Authors:** Lu Jia, Qing Liu, Huiqing Hou, Guangli Guo, Ting Zhang, Songli Fan, Li Wang

**Affiliations:** 1 Department of Obstetrics and Gynecology, Hebei General Hospital, Hebei Medical University, No. 348 Heping Road, Shijiazhuang, 050051 Hebei China; 2 Zibo Maternal and Child Health Hospital, No. 66, North Tianjin Road, Zibo, 255000 Shandong China; 3 Hebei Women and Children’s Health Center, No. 147, Jianhua Street, Shijiazhuang, 050000 China

**Keywords:** Preeclampsia, Air pollution, PM_2.5_, PM_10_, NO_2_, SO_2_, CO, O_3_, Epidemiology, China

## Abstract

**Background:**

Ambient air pollution is becoming a serious environmental problem in China. The results were inconsistent on that air pollution was a risk factor of preeclampsia in pregnancy.

**Methods:**

Total 116,042 pregnant women were enrolled from 22 hospitals in 10 cities of Hebei Province, China from January 1, 2015 to December 31, 2017. The parturients were divided into preeclampsia group (PE group) and non-preeclampsia group (non-PE group). The data of air pollutants, namely, particulate matter (PM)2.5, PM_10_, NO_2_, SO_2_, CO, O_3_ were collected from China Environmental Inspection Station.

**Results:**

Among the 116,042 pregnant women, 2988 (2.57%) pregnant women were diagnosed with preeclampsia. The concentrations of exposed PM_2.5_, PM_10_, NO_2_ and O_3_ in the PE group were significantly higher than those in the non-PE group, and they were risk factors of the PE group in the first and second trimester of pregnancy respectively. The concentrations of exposed SO_2_ and CO in PE patients and non-PE women were not different, but high concentration of these air pollutants were risk factors to PE in the second trimester.

**Conclusion:**

The exposure to PM_2.5_, PM_10_, NO_2_, O_3_ were risk factors for preeclampsia in the first and second trimester of pregnancy, while only at high level, SO_2_ and CO were risk factors for preeclampsia in the second trimester of pregnancy.

## Background

With the development of the economy, ambient air pollution has become a serious environmental problem. And people are more and more concerned about the effects of air quality on health. Studies have shown that air pollution can lead to serious harm to the cardiovascular system and respiratory system [1], and even increase the risk of cancer. Pregnancy is a special stage of life, during which the pregnant women are susceptible to various physical and chemical factors. In recent years, more and more researchers paid attention to the adverse pregnancy outcomes caused by air pollution. For example, air pollutants would increase the risk of preterm birth [2], low birth weight [3], fetal growth restriction [4], and hypertensive disorders during pregnancy [5]. Preeclampsia is one of the most common complications of pregnancy, characterized by hypertension and proteinuria after 20 weeks of gestation. Preeclampsia had many adverse outcomes, such as pulmonary, renal insufficiency, cerebral disturbance, impaired liver function [6, 7], and increased risk of cardiovascular, cerebrovascular diseases, such as coronary heart disease, stroke in the future [8, 9]. Their offsprings were also affected [10]. The incidence rate of preeclampsia varied in the world, with 5.1% in Canada [11], 3.4% in the United States [12], 2.3% in Australia [13], 2.3% in Switzerland [14], 1.3% in Jordan [15]. The incidence rate of preeclampsia in China was about 3.1% in 2011 [16]. Most studies focused on the effects of short-term exposure to air pollutants on the incidence of preeclampsia, but the adverse effects of long-term exposure tended to be greater [17], and the exposure to different air pollutants at different trimesters of pregnancy had different impacts on the morbidity of preeclampsia [18]. There was no unified conclusion about it. A study showed that women exposed to high concentration of traffic pollution during pregnancy increased the risk of preeclampsia by 12% [18]. But another study found that the risk of preeclampsia was not related with the exposure to air pollutants (Particulate Matter (PM), NO_2_, SO_2_, CO, O_3_) during pregnancy [19]. With the rapid development of industry, combined with its special geographic and climate characteristics, North China has become an area with the worst air quality in the world. The objective of this study was to analyze the data of 22 hospitals from Hebei Maternal Near Miss Surveillance System (HBMNMSS) in Hebei Province from 2015 to 2017, and assess the effects of exposure to ambient air pollutants (PM_2.5_, PM_10_, NO_2_, SO_2_, CO, O_3_) on preeclampsia at different trimesters of pregnancy.

## Methods

All participants of this study signed an informed consent, and the protocol was approved by the ethic committee of Hebei Women and Children’s Health Center.

### Data collection

**Fig. 1:**
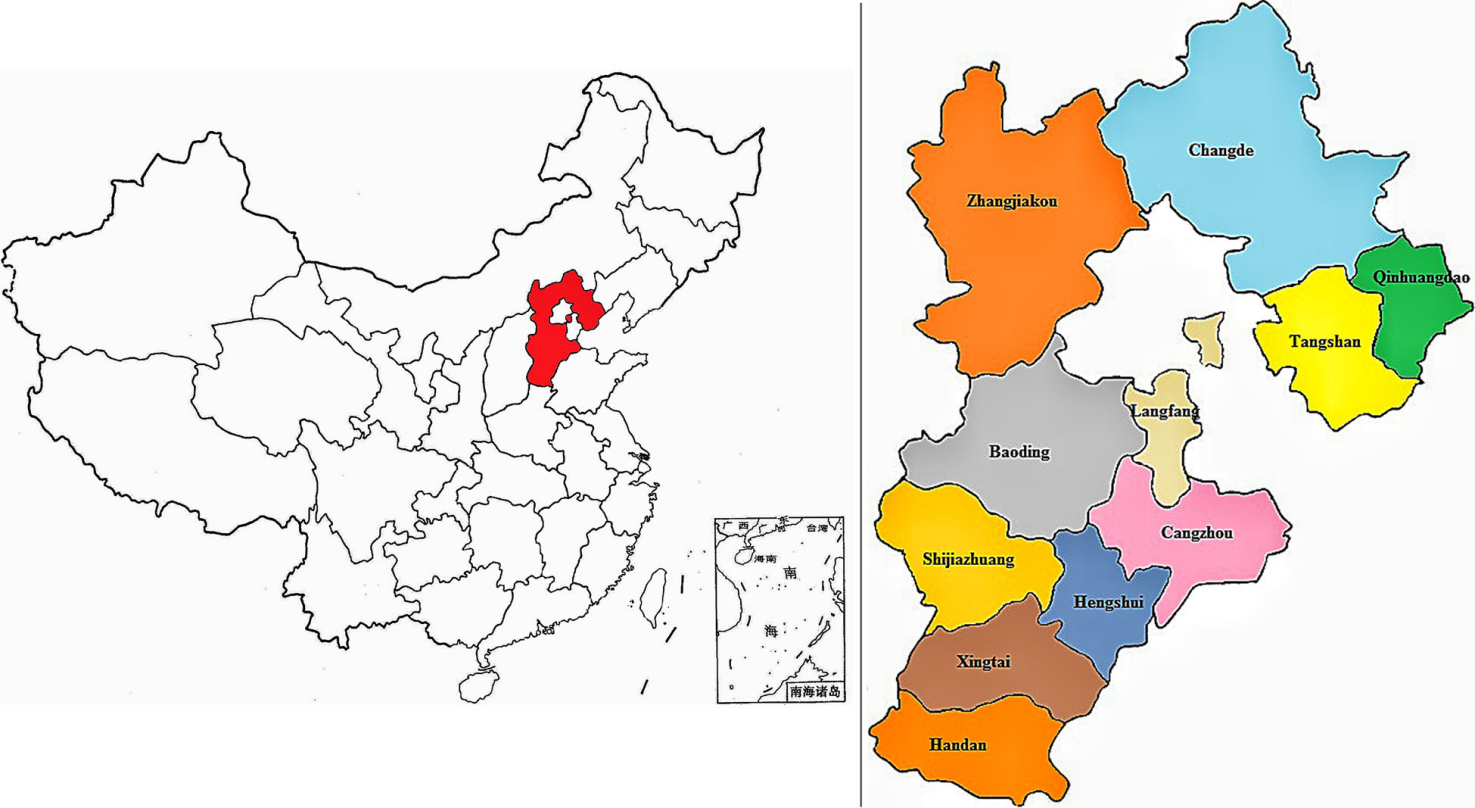
Geographical distribution of Hebei Province

**Fig. 2:**
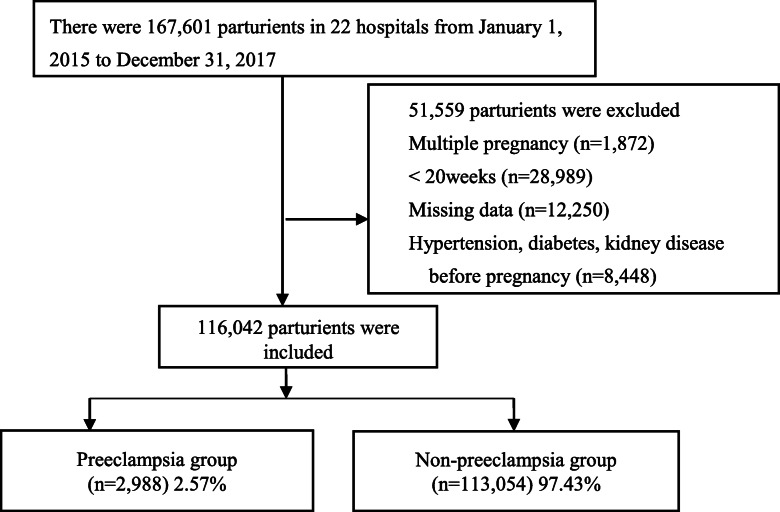
The selection flowchart of participants. The data was collected from Hebei Maternal Near Miss Surveillance System, which involved 22 hospitals in Hebei Province, China. There were 167,601 parturient who gave birth in these hospitals from January 1, 2015 to December 31, 2017. Women with ≥20 gestational weeks, singleton pregnancies were included. Total 116,042 parturients were included in this study

The data was collected from HBMNMSS, which involved 22 hospitals randomly cluster sampled in 10 cities (Shijiazhuang, Baoding, Handan, Zhangjiakou, Chengde, Qinhuangdao, Xingtai, Tangshan, and Hengshui) of Hebei Province, China (Fig. 1). More than 1000 babies deliver in each of the hospitals every year. Total 167,601 parturients were collected from January 1, 2015 to December 31, 2017. The inclusion criteria included singleton pregnancy, with more than 20 gestational weeks; The exclusion criteria included missing data, previous hypertension, diabetes mellitus, nephropathy. Total 116,042 parturients were enrolled, and the selection flowchart of the participants was shown in Fig. 2.

There were six ambient air pollutants in our study, namely, particulate matter (PM_2.5_, PM_10_), nitrogen dioxide (NO_2_), sulfur dioxide (SO_2_), carbon monoxide (CO), ozone (O_3_). Exposure to air pollutants was analyzed with a modified version of the three dimensional multipollutant regional air quality model developed by the US Environmental Protection Agency, the Community Multiscale Air Quality model (version 4.7.1). The data of air pollutants (PM_2.5_, PM_10_, NO_2_, SO_2_, CO and O_3_) were collected from January 2014 to December 2017 from China Environmental Inspection Station (available in http://www.cnemc.cn/). The air quality report of the website included the monthly air quality levels in different cities, and the level of each pollutant. Based on the last menstrual period of pregnant women, the pollution exposure level of each pregnant woman could correspond to different pregnancy months. We take the average exposure concentration of 3 months as the exposure level of pollutants in the first, second and third trimester of pregnancy.

### Definition of variables

Maternal characteristics including gravidity, parity, age, education level, marital status, previous hypertension, diabetes mellitus and nephropathy were collected from the records of the system. Maternal age was divided into 6 subgroups (< 20, 20–24, 25–29, 30–34, 35–39, ≥40 years). Gravidity was stratified into 1, ≥2. Parity was stratified into 0, 1, and ≥ 2. Education level was stratified into three subgroups (college and above, middle school, primary school and below). Hospitals were divided into tertiary hospitals, secondary hospitals, primary hospitals. Seasons were divided into warm season (from May to October) and cool season (from November to April) seasons. The exposure to air pollutants was a cumulative process, the exposure of pregnant women to air pollutants was calculated based on the prevailing international rules, with an assumption that residence area of the women was relatively fixed.

The individual exposure level of air pollution was reflected by monthly average level. The conception date was calculated according to the last menstrual period (LMP), or according to gestational week. If LMP was during the period of 1st to15th day of a month, then this month was defined as the first month of pregnancy; if LMP was during the period of 16th to 31st day of a month, then the next month was defined as the first month of pregnancy. The exposure to air pollutants was collected on a monthly basis. The first to the third month of pregnancy was defined as the first trimester of pregnancy, the fourth to sixth month of pregnancy was defined as the second trimester of pregnancy, and the seventh to ninth month of pregnancy was defined as the third trimester of pregnancy. Because some patients with preeclampsia terminated pregnancies before the third trimester of pregnancy, we did not take the exposure to the air pollutants in the third trimester into consideration in our study.

### Statistical analysis

The parturients were divided into preeclampsia group (PE group) and non- preeclampsia group (non-PE group). Data description was presented as means ± standard deviations or median with interquartile ranges (M (IQR)) for continuous variables and percentages for categorical variables. It was analyzed using One-way ANOVA for normally distributed variables and Kruskal-Wallis H test for non-normally distributed variables. Chi-square test was used in categorical variables. The exposure levels of air pollutants were categorical variables, and nonparametric rank sum test was used. Odds ratio (OR) with corresponding 95% confidence intervals (CI) were calculated to explore the associations between air pollutants and preeclampsia by univariate and multivariate logistic regression analysis. Stratified analysis was conducted based on the first and the second trimester of pregnancy. In the unadjusted model (univariate logistic regression analysis), preeclampsia was taken as a dependent variable, and exposure level of air pollutants was taken as an independent variable. In adjusted model, trimester, gravidity, parity, maternal age, maternal education, hospital level, conception and delivery season was adjusted. In order to analyze the effects of pollutants on the incidence of preeclampsia, the concentration of pollutants was stratified into four groups according to the quartile (Q1, Q2, Q3, Q4). The logistic regression analysis was performed with Q1 as the reference. All *P* values were two-sided, with *P* <  0.05 considered statistically significant. All analyses were performed using IBM SPSS 21.0.

## Results

### Characteristics of parturients

Total 116,042 parturients were included, and 2988 (2.57%) women were diagnosed with preeclampsia. There were significant differences between parity, maternal age, and maternal education in the two groups, but there were no significant differences between PE group and non-PE group in gravidity, marital status, and season of conception. The ratio of parturients with PE was higher in nulliparous women (47.3%), multiparous women with parity ≥2 (7.4%), and parturients with age < 20 years (1.6%) or ≥ 35 years (14.2%), lower education, premature delivery than that of parturients without PE. The maternal and perinatal characteristics of the parturients were shown in Table 1.

**Table 1:** Maternal and Perinatal Baseline Characteristics

	PE group (***N*** = 2988)	non-PE group (***N*** = 113,054)	***χ***^***2***^	***P***
Gravidity			1.437	0.231
1	1089 (36.4)	40,002 (35.4)		
≥ 2	1899 (63.6)	73,052 (64.6)		
Parity			40.137	< 0.001
0	1413 (47.3)	49,479 (43.8)		
1	1355 (45.3)	57,234 (50.6)		
≥ 2	220 (7.4)	6341 (5.6)		
Age (years)			176.188	< 0.001
< 20	47 (1.6)	1304 (1.2)		
20–24	472 (15.8)	18,094 (16.0)		
25–29	1201 (40.2)	53,034 (46.9)		
30–34	753 (25.2)	28,463 (25.2)		
35–39	406 (13.6)	10,451 (9.2)		
≥ 40	109 (3.6)	1708 (1.5)		
Education level			84.782	< 0.001
College and above	956 (32.0)	41,755 (36.9)		
Middle school	1921 (64.3)	69,307 (61.3)		
Primary school and below	111 (3.7)	1992 (1.8)		
Marital status			0.272	0.602
Married	2974 (99.5)	112,594 (99.6)		
Single or divorced	14 (0.5)	460 (0.4)		
Season of conception			0.134	0.714
Warm (May to October)	1396 (46.7)	53,202 (47.1)		
Cold (November to April)	1592 (53.3)	59,852 (52.9)		
Season of delivery			3.668	0.160
Warm (May to October)	1304 (43.6)	48,700 (43.1)		
Cold (November to April)	1361 (45.5)	53,111 (47.0)		
Gestational age (weeks)			5201.901	< 0.001
≥ 37	1834 (61.4)	106,660 (94.3)		
< 37	1154 (38.6)	6394 (5.7)		

Total 116,042 parturients were included. The categorical data were presented as numbers (frequencies, %), Chi-square test was used for categorical data. *PE* preeclampsia

### Exposure to air pollutants

The concentration of exposed PM_2.5_, PM_10_, NO_2_, SO_2_, CO, O_3_ in the first and second trimesters of pregnancy was shown in Table 2. The concentrations of exposed PM_2.5_, PM_10_, NO_2_ and O_3_ in PE group were higher than those in non-PE group in both two trimesters (*P* <  0.05). There was no significant difference between the two groups in the concentration of exposed SO_2_ and CO (*P* > 0.05).

**Table 2:** Concentration of Exposure to Air Pollutants in the First and Second Trimester of Pregnancy

	PE group (***N*** = 2988)	non-PE group (***N*** = 113,054)	***Z***	***P***
PM_2.5_(μg/m^3^)				
Trimester 1	72.67 (61.33, 111.67)	71.33 (55.67, 107.67)	−6.818	< 0.001
Trimester 2	70.67 (58.33, 106.67)	68.67 (54.67, 99.67)	−7.424	< 0.001
PM_10_(μg/m^3^)				
Trimester 1	139.00 (107.67, 181.33)	135.33 (101.00, 169.67)	−7.293	< 0.001
Trimester 2	135.00 (106.67, 169.67)	134.67 (99.00, 160.67)	−6.464	< 0.001
NO_2_ (μg/m^3^)				
Trimester 1	52.33 (40.00, 63.33)	51.67 (39.67, 63.33)	−2.753	0.006
Trimester 2	50.67 (39.67, 64.00)	50.67 (39.67, 63.33)	−3.248	0.001
SO_2_ (μg/m^3^)				
Trimester 1	37.33 (23.00, 53.67)	36.67 (23.33, 53.67)	−1.344	0.179
Trimester 2	33.33 (21.67, 50.33)	33.33 (22.00, 49.00)	−2.036	0.052
CO (mg/m^3^)				
Trimester 1	2.23 (1.40, 3.87)	2.37 (1.50, 3.80)	−1.300	0.194
Trimester 2	2.10 (1.40, 3.87)	2.13 (1.40, 3.70)	−0.545	0.586
O_3_ (μg/m^3^)				
Trimester 1	132.67 (86.00, 174.67)	126.00 (81.67, 170.33)	−5.130	< 0.001
Trimester 2	145.67 (86.00, 182.00)	135.67 (82.67, 180.67)	−2.481	0.013

The concentration of exposure to air pollutants were presented as median (interquartile ranges); The difference was analyzed using One-way ANOVA for normally distributed variables and Kruskal-Wallis H test for non-normally distributed variables. *Trimester 1* the first trimester of pregnancy, *Trimester 2* the second trimester of pregnancy, *PE* preeclampsia

### Effect of ambient air pollutants on preeclampsia

After adjusted trimester, gravidity, parity, maternal age, maternal education, hospital level, conception and delivery season, exposure to PM_2.5_ was an independent risk factor for preeclampsia, and the OR (95% CI) were 1.014 (1.000–1.028) and 1.026 (1.012–1.049) in the first and second trimester of pregnancy respectively, and with the increased concentration of PM_2.5_, the OR value increased gradually. Exposure to PM_10_ was also an independent risk factor for preeclampsia, and the OR (95% CI) were 1.013 (1.002–1.023) and 1.016 (1.005–1.027) in the first and second trimester of pregnancy respectively, and with the increased concentration of PM_10_, the OR value increased in the first trimester of pregnancy, but not in the second trimester. The exposure to NO_2_ was an independent risk factor for preeclampsia, and the OR (95% CI) were 1.046(1.003–1.091) and 1.056 (1.010–1.105) in the first and second trimester respectively, and with the increased concentration of NO_2_, the OR value increased in the first trimester of pregnancy, but was not correlated with preeclampsia in the second trimester of pregnancy. The exposure to SO_2_ was an independent risk factor for preeclampsia, and the OR (95% CI) was 1.013 (0.985–1.068) and 1.050 (1.019–1.082) in the first and second trimester of pregnancy respectively. There was no significant correlation between SO_2_ exposure and preeclampsia in first trimester. While in the second trimester, only in the Q3 and Q4 subgroups, it was a risk factor for preeclampsia, and the OR (95% CI) was 1.164 (1.022–1.326) and 1.317 (1.119–1.551). The exposure to CO was an independent risk factor for preeclampsia, the OR (95% CI) was 1.297(0.910–1.849) and 2.305(1.602–3.317) in the first and second trimester of pregnancy respectively. But only in Q3 and Q4 subgroups in the second trimester, it was a risk factor for preelampsia, the OR (95% CI) was 1.160 (1.016–1.324) and 1.238 (1.038–1.475). The risk of preeclampsia also increased with O_3_ exposure, the OR (95% CI) was 1.024 (1.010–1.037) and 1.016 (1.003–1.029) in the first and second trimester of pregnancy respectively. With the increase of exposed concentration of O_3_, the risk of preeclampsia increased in the first trimester. The results were shown in Table 3.

**Table 3:** Analysis of Risk Factors for Preeclampsia in the First and Second Trimester of Pregnancy

EC	*N* (PE/Total)	Unadjusted Model		Adjusted Model	
		OR (95% CI)	*P*	AOR (95% CI)	*P*
**PM**_**2.5**_					
Trimester 1		1.027 (1.018–1.037)	< 0.001	1.014 (1.000–1.028)	0.005
Q 1	553/28225	Reference		Reference	
Q 2	856/29721	1.541 (1.381–1.719)	< 0.001	1.254 (1.120–1.405)	< 0.001
Q 3	786/29102	1.442 (1.290–1.612)	< 0.001	1.225 (1.081–1.388)	0.001
Q 4	813/28994	1.499 (1.342–1.647)	< 0.001	1.235 (1.064–1.434)	0.006
Trimester 2		1.034 (1.024–1.043)	< 0.001	1.026 (1.012–1.049)	< 0.001
Q 1	570/ 29,262	Reference		Reference	
Q 2	814/ 29,033	1.452 (1.303–1.618)	< 0.001	1.173 (1.038–1.325)	0.010
Q 3	773/ 28,830	1.387 (1.243–1.547)	< 0.001	1.179 (1.039–1.338)	0.011
Q 4	831/ 28,917	1.489 (1.337–1.659)	< 0.001	1.324 (1.142–1.535)	< 0.001
**PM**_**10**_					
Trimester 1		1.021 (1.014–1.028)	< 0.001	1.013 (1.002–1.023)	0.021
Q 1	550/29364	Reference		Reference	
Q 2	781/28742	1.463 (1.310–1.634)	< 0.001	1.269 (1.131–1.423)	< 0.001
Q 3	862/29440	1.580 (1.418–1.761)	< 0.001	1.401 (1.231–1.594)	< 0.001
Q 4	795/28496	1.504 (1.347–1.678)	< 0.001	1.429 (1.228–1.664)	< 0.001
Trimester 2		1.024 (1.017–1.031)	< 0.001	1.016 (1.005–1.027)	< 0.001
Q 1	602/29152	Reference		Reference	
Q 2	779/28350	1.340 (1.203–1.492)	< 0.001	1.098 (0.982–1.228)	0.099
Q 3	811/29599	1.336 (1.201–1.486)	< 0.001	1.163 (1.020–1.326)	0.024
Q 4	796/28941	1.341 (1.205–1.493)	< 0.001	1.094 (0.950–1.260)	0.201
**NO**_**2**_					
Trimester 1		1.040 (1.016–1.066)	0.001	1.046 (1.003–1.091)	0.034
Q 1	668/28909	Reference		Reference	
Q 2	793/29415	1.171 (1.055–1.300)	0.003	1.159 (1.034–1.299)	0.011
Q 3	796/30061	1.150 (1.036–1.276)	0.009	1.223 (1.071–1.396)	0.003
Q 4	731/27657	1.148 (1.032–1.276)	< 0.011	1.292 (1.101–1.512)	0.002
Trimester 2		1.054 (1.029–1.080)	< 0.001	1.056 (1.010–1.105)	0.017
Q 1	784/ 30,347	Reference		Reference	
Q 2	723/ 28,503	0.981 (0.886–1.087)	0.719	0.890 (0.796–0.994)	0.039
Q 3	661/ 28,049	0.910 (0.820–1.011)	0.078	0.894 (0.779–1.025)	0.110
Q 4	820/ 29,143	1.092 (0..989–1.206)	0.083	0.934 (0.791–1.103)	0.421
**SO**_**2**_					
Trimester 1		1.012 (0.994–1.030)	0.204	1.013 (0.985–1.068)	0.361
Q 1	761/29302	Reference		Reference	
Q 2	713/28691	0.956 (0.862–1.060)	0.392	1.024 (0.908–1.156)	0.698
Q 3	769/29332	1.010 (0.912–1.118)	0.852	1.051 (0.919–1.201)	0.469
Q 4	745/28717	0.999 (0.902–1.107)	0.983	0.954 (0.804–1.131)	0.588
Trimester 2		1.021 (1.002–1.041)	0.031	1.050 (1.019–1.082)	0.002
Q 1	752/ 28,886	Reference		Reference	
Q 2	711/ 29,067	0.938 (0.846–1.041)	0.228	1.059 (0.937–1.198)	0.357
Q 3	741/ 29,978	0.948 (0.856–1.051)	0.310	1.164 (1.022–1.326)	0.022
Q 4	784/ 28,111	1.073 (0.970–1.188)	0.171	1.317 (1.119–1.551)	0.001
**CO**					
Trimester 1		0.943 (0.743–1.195)	0.625	1.297 (0.910–1.849)	0.151
Q 1	792/28820	Reference		Reference	
Q 2	780/30067	0.943 (0.853–1.042)	0.247	1.084 (0.965–1.218)	0.172
Q 3	640/28112	0.824 (0.742–0.916)	< 0.001	1.083 (0.947–1.237)	0.245
Q 4	776/29043	0.972 (0.879–1.074)	0.573	1.139 (0.969–1.338)	0.114
Trimester 2		1.305 (1.034–1.648)	0.025	2.305 (1.602–3.317)	< 0.001
Q 1	844/ 30,607	Reference		Reference	
Q 2	652/ 27,083	0.870 (0.784–0.965)	0.008	1.019 (0.904–1.149)	0.759
Q 3	679/ 28,681	0.855 (0.772–0.947)	0.003	1.160 (1.016–1.324)	0.029
Q 4	813/ 29,671	0.993 (0.901–1.095)	0.896	1.238 (1.038–1.475)	0.017
**O**_**3**_					
Trimester 1		1.021 (1.013–1.029)	< 0.001	1.024 (1.010–1.037)	0.001
Q 1	653/ 28,869	Reference		Reference	
Q 2	726/ 29,512	1.090 (0.979–1.213)	0.115	1.205 (1.052–1.382)	0.007
Q 3	816/ 29,378	1.234 (1.112–1.370)	< 0.001	1.324 (1.136–1.544)	< 0.001
Q 4	793/ 28,283	1.246 (1.122–1.384)	< 0.001	1.334 (1.132–1.574)	0.001
Trimester 2		1.010 (1.003–1.017)	0.006	1.016 (1.003–1.029)	0.013
Q 1	732/ 29,538	Reference		Reference	
Q 2	712/ 28,467	1.010 (0.909–1.121)	0.859	1.214 (1.072–1.375)	0.002
Q 3	756/ 29,362	1.040 (0.938–1.153)	0.455	1.177 (1.011–1.369)	0.035
Q 4	788/ 28,675	1.112 (1.004–1.231)	0.041	1.239 (1.035–1.483)	0.020

There were 113,054 parturients in Non-PE group, and 2988 parturients in PE group. The concentration of exposed ambient air pollutants (PM_2.5_, PM_10_, NO_2_, SO_2_, CO, O_3_) was stratified into four groups according to the quartile (Q1, Q2, Q3, Q4); Q1 acted as the reference, univariate logistic regression was used to examine the strength of association between exposure to air pollutants and preeclampsia; In adjusted model, trimester, gravidity, parity, age, education, hospital level, conception and delivery season were adjusted. *PE* preeclampsia, *OR* Odds ratio, *CI* confidence intervals, *Trimester 1* the first trimester of pregnancy, *Trimester 2* the second trimester of pregnancy

## Discussion

The effects of ambient air pollution on human health have attracted increasing concern in the world. Hebei Province is located in the east of China. The western part of Hebei Province is a mountainous area, the northern part is a plateau, while the central part is the North China Plain, where the ambient air pollution in autumn and winter is very serious. The main pollutants were PM_2.5_, PM_10_, NO_2_, SO_2_, CO and O_3_ according to the air quality report of China’s Environmental Monitoring. The data from China Environmental Quality Monthly Report showed that there were obvious regional differences in the air pollution level in Hebei Province. The cities with serious air pollution included Baoding, Shijiazhuang, Handan and other cities on the North China Plain. The cities with less serious air pollution were Zhangjiakou, Chengde, Qinhuangdao and other mountainous and coastal cities.

There were 2988 patients diagnosed with preeclampsia in 116,042 deliveries in 22 hospitals in HBMNMSS, from 2015 to 2017. The incidence rate of preeclampsia was 2.57%, which was lower than that published in some other related reports [6], this may be associated with the exclusion of twins pregnancy, previous hypertension, diabetes, kidney disease, and other high-risk factors in the study. Beside the risk factors for preeclampsia such as nulliparous women, adolescent pregnancy, less education years, cold season [7, 9], our study also confirmed that ambient air pollutants were independent risk factors for preeclampsia. The concentrations of exposed PM_2.5_, PM_10_, NO_2_ and O_3_ in PE patients were higher than those in non-PE women, and the exposure to them were risk factors for preeclampsia. The concentrations of SO_2_ and CO exposure in PE patients and non-PE women were not different, but at higher concentration, SO_2_ and CO exposure were also risk factors for preeclampsia in the second trimester.

There were many theories on the mechanism that air pollutants have effects on preeclampsia. For the pregnant women, exposure to air pollution during early pregnancy might lead to a decrease in oxygen exchange in their lungs, therefore, the tissue cells were in an oxygen-deficient environment, which induced the proliferation of trophoblasts. The oxidative stress was reduced to protect trophoblasts from DNA damage and to inhibit the invasion of trophoblasts [20]. This would cause the insufficient recasting of uterine spiral arterioles, and shallow implantation of placenta, which was one of the pathogenesis of preeclampsia [21]. Air pollutants or respirable solid particles would enter the blood circulation system, induce systemic oxidative stress, promote the release of inflammatory factors, lead to vascular endothelial dysfunction, autonomic nervous system imbalance and vasoconstriction [22, 23]; At the same time, oxidative stress and inflammation could induce increased sympathetic activity, arterial remodeling, and elevated blood pressure [24]. Studies have shown that the pregnant women exposing to air pollutants can cause insulin resistance, lead to hyperinsulinemia, reduce the NO synthesis and abnormal lipid metabolism, affect the synthesis of prostaglandin E_2_, increase peripheral vascular resistance, and result in elevated blood pressure [25].

The exposure level of PM_2.5_ in PE patients was higher than that in non-PE women in the first and second trimester. With the increase of concentration of PM_2.5_ exposure by every 10 μg/m^3^, the risk of preeclampsia increased by 1.014 and 1.026 times in the first and second trimester of pregnancy. Chen found that the risk of hypertension increased by 1.130 (1.052–1.221) times with the increase of concentration of PM_2.5_ exposure by every 10 μg/m^3^ [26]. A cohort study reported by Lee also showed an increased risk of preeclampsia in pregnant women by 15% with the increase of concentration of PM_2.5_ exposure by every 4 μg/m^3^ during the first trimester of pregnancy [27]. Pedersen et al. showed an increase in risk of preeclampsia in pregnant women by 1.312 (1.140–1.503) times with the increase of concentration of PM_2.5_ exposure by every 5 μg/m^3^ through a meta-analysis of 17 studies [28]. However, Rudra et al. showed no significant association between PM_2.5_ exposure and preeclampsia in a prospective study in Washington State [29].

PM_10_ exposure level in PE patients was also higher than that in non-PE women. The risk of preeclampsia increased by 1.013 and 1.016 times respectively with the increase of concentration of PM_10_ exposure by 10 μg/m^3^ in the first and the second trimester of pregnancy. Other study showed an increased 1.11 mmHg in systolic blood pressure with the increase of concentration of PM_10_ exposure by 10 μg/m^3^ in the second trimester [30], but during the first trimester the PM_10_ exposure does not rule out its hysteresis. A cohort study for in the United States reported that with an increase of concentration of PM_10_ exposure by 3.92 μg/m^3^, the risk for gestational hypertension increased by 1.072 times [31]. However, a study in Korea showed no significant association between PM_10_ exposure and preeclampsia [32].

The concentration of exposure to NO_2_ in PE patients was higher than that of non-PE women. For every 10 μg/m^3^ increase of NO_2_ exposure during the first and the second trimester, the risk of preeclampsia increased by 1.046 and 1.056 times. In the first trimester of pregnancy, the risk of preeclampsia increased with the increase of concentration of NO_2_ exposure. A meta-analysis by Hu et al. showed that for every 10 ppb increase of NO_2_ exposure during pregnancy, the risk of preeclampsia increased by 1.101 (1.032–1.170) times [33]. The Hooven’s study also found that exposure to NO_2_ was associated with increased systolic blood pressure in the first and second trimester of pregnancy [30]. A study from Norway showed that there was no significant correlation between low level of NO_2_ exposure during pregnancy and the risk of preeclampsia [34], which was consistent with the result that only the highest exposure to NO_2_ was the risk factor for preeclampsia in the second trimester of pregnancy, and this conclusion was consistent with our study [35].

Although the concentration of SO_2_ exposure increased every 10 μg/m^3^ in the first and the second trimester, the risk of preeclampsia increased by 1.013 and 1.050 times respectively. However, only the highest exposure to SO_2_ was an independent risk factor for preeclampsia in the second trimester. Few studies have analyzed the correlation between SO_2_ exposure and preeclampsia. A study in Japan showed that the outcome between SO_2_ and hypertensive disorder in pregnancy was not invariable [36]. There was no significant correlation between SO_2_ and hypertensive disorder in pregnancy in Choe’s study, but the severity of the disease increased with the increase of SO_2_ exposure [32]. Cai’s study showed that long-term exposure to SO_2_ was also positively correlated with hypertension, but there was no statistical significance [37]. Further study needed to be done.

With the increase of concentration of CO by every 10 mg/m^3^ in the first and second trimester, the risk of preeclampsia increased by 1.486 and 2.858 times, but only at a higher concentration of exposure, CO was an independent risk factor for preeclampsia, and the CO exposure of moderate concentration was an protective factor for preeclampsia, which suggested a bidirectional effect of CO on preeclampsia. A study from Canada also showed that CO exposure of moderate concentration was an independent protective factor for preeclampsia [38]. CO was considered to increase trophoblast invasion, blood flow to the uterus placenta, to reduce hypoxia-induced apoptosis, and to up-regulated the anti-oxidant system of the placenta [39]. At present, there was obvious controversy about the correlation between CO and preeclampsia. Hu’s study showed that for every 1 ppm increase in the concentration of CO exposure in first trimester of pregnancy, the risk of hypertensive disorder pregnancy increased by 1.791 (1.312–2.450) times [33]. The cross-sectional study of Quinn showed that for every 1 ppm increase in the concentration of CO exposure, the diastolic blood pressure increased 0.43 mmHg [40]. However, other studies have also found that exposure to CO during pregnancy could reduce the risk of preeclampsia, and there was a dose-response relationship between them [38].

Patients with preeclampsia had higher concentration of O_3_ exposure during pregnancy. With the increase of the concentration of O_3_ exposure by every 10 μg/m^3^ during the first trimester, the risk of preeclampsia increased by 1.024 times, the higher the concentration of O_3_ exposure, the higher the risk of preeclampsia, the risk was shown in a Japanese study [36] and other studies [33, 35, 41] in other regions of the world.

### Strengths and limitations

The study covered the parturients in 10 cities in Hebei Province, with large sample size and representative population. We confirmed that all 6 pollutants including PM_2.5_, PM_10_, NO_2_, SO_2_, CO and O_3_ were risk factors for preeclampsia, the risks increased with the increase of concentration of PM_2.5_, PM_10_, NO_2_, O_3_. However, only at high level, SO_2_ and CO were risk factors for preeclampsia in the second trimester. This study was a retrospective study, the environmental data was derived from environmental monitoring sites, and was not measured at individual exposure concentration, the exact exposure to air pollution for each women was not very clear, which may limit our accuracy in assessing the correlation between air pollutants and preeclampsia. The interactive effects of six pollutants on preeclampsia were not analyzed. The data of smoking status, living habits, family history of preeclampsia, etc. was absent. The ambient air pollution is very severe in Hebei Province, which is much higher than that in Europe and the United States, so its effect on preeclampsia may be overestimated or underestimated.

## Data Availability

The datasets used and/or analyzed during the current study are available from the corresponding author on reasonable request.

## References

[1] Liu W, Huang C, Hu Y, Fu Q, Zou Z, Sun C, Shen L, Wang X, Cai J, Pan J, Huang Y, Chang J, Sun Y (2016). J., S., associations of gestational and early life exposures to ambient air pollution with childhood respiratory diseases in Shanghai, China: a retrospective cohort study. Environ Int.

[2] Wang Q, Benmarhnia T, Zhang H, Knibbs LD, Sheridan P, Li C, Bao J, Ren M, Wang S, He Y, Zhang Y, Zhao Q, C H (2018). Identifying windows of susceptibility for maternal exposure to ambient air pollution and preterm birth. Environ Int.

[3] Gong X, L. Y, Bell ML, Zhan FB (2018). Associations between maternal residential proximity to air emissions from industrial facilities and low birth weight in Texas, USA. Environ Int.

[4] Nobles CJ, Grantz KL, Liu D, Williams A, Ouidir M, Seeni I, Sherman S, Mendola P (2019). Ambient air pollution and fetal growth restriction: physician diagnosis of fetal growth restriction versus population-based small-for-gestational age. Sci Total Environ.

[5] Zahra M, Salam MT, Murphy, G T, Frederick L, Ingles SA, Wilson ML (2013). Associations between ambient air pollution and hypertensive disorders of pregnancy. Environ Res.

[6] WJ, M. B.; T, R. C.; Shakila, T.; a, M. L.; De, G. C. J. M.; Justus, H. G (2016). Pre-eclampsia. Lancet.

[7] American college of obstetricians and gynecologist; Pregnancy, T. F. o. H. i., Report of the American College of Obstetricians and Gynecologists′ Task Force on Hypertension in Pregnancy. Obstet Gynecol. 2013;122(5):1122–31. 10.1097/01.AOG.0000437382.03963.88 24150027

[8] Barnes JN, Harvey RE, Miller KB, Jayachandran M, BD MKRL, Bailey KR, Joyner MJ, VM. M (2018). Cerebrovascular reactivity and vascular activation in postmenopausal women with histories of preeclampsia. Hypertension.

[9] Mulder EG, Ghossein-Doha C, Froeling MFEM, van Kuijk SMJ, MEA. S (2018). Recurrence rates of preeclampsia over the past 20 years in women assessed for non-pregnant cardiovascular risk factors. Pregnancy Hypertension.

[10] Sacks KN, Friger M, Shoham-Vardi I, Spiegel E, Sergienko R, Landau D, Sheiner E (2018). Prenatal exposure to preeclampsia as an independent risk factor for long- term cardiovascular morbidity of the offspring. Pregnancy Hypertension.

[11] Auger N, Luo ZC, Nuyt AM, Kaufman JS, Naimi AI, Platt RW, WD. F (2016). Secular Trends in Preeclampsia Incidence and Outcomes in a Large Canada Database: A Longitudinal Study Over 24 Years. Canadian J Cardiol.

[12] V., A. C.; M., K. K.; J., W. R (2013). Pre-eclampsia rates in the United States, 1980–2010: age-period-cohort analysis. BMJ.

[13] Thornton C, Dahlen H, Korda A, A. H (2013). The incidence of preeclampsia and eclampsia and associated maternal mortality in Australia from population-linked datasets: 2000–2008. Am J Obstet Gynecol.

[14] Purde MT, Baumann M, Wiedemann U, Nydegger UE, Risch L, Surbek D, M. R (2015). Incidence of preeclampsia in pregnant Swiss women. Swiss Med Wkly.

[15] Khader YS, Batieha A, Al-Njadat RA, Hijazi SS (2018). Preeclampsia in Jordan: incidence, risk factors, and its associated maternal and neonatal outcomes. J Matern Fetal Neonatal Med.

[16] Ye C, Ruan Y, Zou L, Li G, Li C, Chen Y, Jia C, Megson IL, Wei J, Zhang W (2014). The 2011 survey on hypertensive disorders of pregnancy (HDP) in China: prevalence, risk factors, complications, pregnancy and perinatal outcomes. PLoS One.

[17] Guan T, Xue T, Gao S, Min Hu d XL, Qiu X, Liu X, Zhu T (2019). Acute and chronic effects of ambient fine particulate matter on preterm births in Beijing, China: A time-series model. Sci Total Environ.

[18] Gavin P, Fatima H, Shand AW, Carol B, Angus C, Natasha N (2013). Association between pre-eclampsia and locally derived traffic-related air pollution: a retrospective cohort study. J Epidemiol Community Health.

[19] Nahidi F, Gholami R, Rashidi Y, Majd HA (2014). Relationship between air pollution and pre-eclampsia in pregnant women: a case-control study. East Mediterr Health J.

[20] Berthold H, Martin G, Kristina O, Julia KN, Gerit M (2010). Oxygen as modulator of trophoblast invasion. J Anat.

[21] Sava RI, March KL, Pepine CJ (2018). Hypertension in pregnancy: taking cues from pathophysiology for clinical practice. Clin Cardiol.

[22] Sanidas E, Papadopoulos DP, Grassos H, Velliou M, Tsioufis K, Barbetseas J, Papademetriou V (2017). Air pollution and arterial hypertension. A new risk factor is in the air. J Am Soc Hypertens.

[23] Ahmed A (2015). W., R., Unravelling the theories of pre-eclampsia: are the protective pathways the new paradigm?. Br J Pharmacol.

[24] Wong Martin CS, Tam Wilson WS, Wang Harry HX, Lao XQ, Dexin ZD, Chan Sky WM, Kwan Mandy WM, Fan Carmen KM, Cheung Clement SK, LH., T. E (2014). Exposure to air pollutants and mortality in hypertensive patients according to demography: a 10 year case-crossover study. Environ Pollut.

[25] Haberzettl P, O'Toole TE, Bhatnagar A, Conklin DJ (2016). Exposure to fine particulate air pollution causes vascular insulin resistance by inducing pulmonary oxidative stress. Environ Health Perspect.

[26] Chen H, Burnett Richard T, Kwong Jeffrey C, Villeneuve Paul J, Goldberg Mark S, Brook Robert D, Donkelaar AV, Jerrett M, Martin Randall V, Kopp A (2014). Spatial association between ambient fine particulate matter and incident hypertension. Circulation.

[27] Talbott, O E, Lee, Pei-Chen, Roberts (2013). Catov, First Trimester Exposure to Ambient Air Pollution, Pregnancy;Complications and Adverse Birth Outcomes in Allegheny County, PA. Matern Child Health J.

[28] Pedersen M, Stayner L, Slama R, Sørensen M, Figueras F, Nieuwenhuijsen MJ, Raaschou-Nielsen O, P. D (2014). Ambient air pollution and pregnancy-induced hypertensive disorders: a systematic review and meta-analysis. Hypertension.

[29] Rudra CB, Williams MA, Lianne S, Koenig JQ, Schiff MA (2011). Ambient carbon monoxide and fine particulate matter in relation to preeclampsia and preterm delivery in western Washington state. Environ Health Perspect.

[30] Hooven EH, Den V, Yvonne DK, Pierik FH, Albert H, Ratingen SWV, Zandveld PYJ, Mackenbach JP, Steegers EAP, Miedema HME, Jaddoe VWV (2011). Air pollution, blood pressure, and the risk of hypertensive complications during pregnancy: the generation R study. Hypertension.

[31] Vinikoor-Imler LC, Gray SC, Edwards SE, ML M (2012). The effects of exposure to particulate matter and neighbourhood deprivation on gestational hypertension. Paediatr Perinat Epidemiol.

[32] Choe SA, Jun YB, SY. K (2018). Exposure to air pollution during preconceptional and prenatal periods and risk of hypertensive disorders of pregnancy: a retrospective cohort study in Seoul, Korea. BMC Pregnancy Childbirth.

[33] Hui H, Sandie H, Jeffrey R, Greg K, Xiaohui X, O., T. E (2014). Ambient air pollution and hypertensive disorders of pregnancy: a systematic review and meta-analysis. Atmos Environ.

[34] Christian M, Eldevik HS, Geir A, Hein S, Per M, Wenche N, Per N, J., L. S (2018). Preeclampsia and hypertension during pregnancy in areas with relatively low levels of traffic air pollution. Matern Child Health J.

[35] Olsson D, Mogren I, Forsberg B (2013). Air pollution exposure in early pregnancy and adverse pregnancy outcomes: a register-based cohort study. BMJ Open.

[36] Takehiro M, Seiichi M, Kotaro F, Kayo U, Ayano T, Kiyoko K, Hiroshi N (2015). A register-based study of the association between air pollutants and hypertensive disorders in pregnancy among the Japanese population. Environ Res.

[37] Cai Y, Zhang B, Ke W, Feng B, Lin H, Xiao J, Zeng W, Li X, Tao J, Yang Z, Ma W (2016). T., L., associations of short-term and long-term exposure to ambient air pollutants with hypertension: a systematic review and meta-analysis. Hypertension.

[38] Desheng Z, Yanfang G, Graeme S, Daniel K, Mark W, Wen. SW (2012). Maternal exposure to moderate ambient carbon monoxide is associated with decreased risk of preeclampsia. Am J Obstet Gynecol.

[39] Harper LM, Keegan MB, McPherson JA, AS. T (2012). Discussion: 'Moderate ambient level of carbon monoxide and risk of preeclampsia' by Zhai et al. Am J Obstet Gynecol.

[40] Quinn AK, Ae-Ngibise KA, Jack DW, Boamah EA, Enuameh Y, Mujtaba MN, Chillrud SN, Wylie BJ, Owusu-Agyei S, Kinney PL (2016). Association of Carbon Monoxide exposure with blood pressure among pregnant women in rural Ghana: evidence from GRAPHS. Int J Hyg Environ Health.

[41] Hu H, Ha S, Xu X (2017). Ozone and hypertensive disorders of pregnancy in Florida: identifying critical windows of exposure. Environ Res.

